# Healthcare workers’ experiences regarding scaling up of training on integrated disease surveillance and response (IDSR) in Uganda, 2016: cross sectional qualitative study

**DOI:** 10.1186/s12913-019-3923-6

**Published:** 2019-02-13

**Authors:** Lydia Nakiire, Ben Masiira, Christine Kihembo, Edson Katushabe, Nasan Natseri, Immaculate Nabukenya, Innocent Komakech, Issa Makumbi, Okot Charles, Francis Adatu, Miriam Nanyunja, Peter Nsubuga, Solomon Fisseha Woldetsadik, Patrick Tusiime, Ali Ahmed Yahaya, Ibrahima Socé Fall, Alemu Wondimagegnehu

**Affiliations:** 1 Public Health Emergency Operation Centre, Ministry of Health, P.O BOX 7072 Kampala, Uganda; 2 Epidemiology and Surveillance Division, Ministry of Health Kampala, Kampala, Uganda; 3 World Health Organization, Country Office, Kampala, Uganda; 4 Ministry of Health, Kampala, Uganda; 5 Global Public Health Solutions Inc, Atlanta, Georgia USA; 6 World Health Organization Africa Regional Office, Brazzaville, Congo; 7 National Disease Control, Ministry of Health, Kampala, Uganda

**Keywords:** Healthcare workers, Surveillance, Training, Experience, Uganda

## Abstract

**Background:**

The Integrated Disease Surveillance and Response (IDSR) strategy was adopted as the framework for implementation of International Health Regulation (2005) in the African region of World Health Organisation (WHO AFRO). While earlier studies documented gains in performance of core IDSR functions, Uganda still faces challenges due to infectious diseases. IDSR revitalisation programme aimed to improve prevention, early detection, and prompt response to disease outbreaks. However, little is known about health worker’s perception of the revitalised IDSR training.

**Methods:**

We conducted focus group discussions of health workers who were trained between 2015 and 2016. Discussions on benefits, challenges and possible solutions for improvement of IDSR training were recorded, transcribed, translated and coded using grounded theory.

**Results:**

In total, 22/26 FGDs were conducted. Participants cited improved completeness and timeliness of reporting, case detection and data analysis and better response to disease outbreaks as key achievements after the training. Programme challenges included an inadequate number of trained staff, funding, irregular supervision, high turnover of trained health workers, and lack of key logistics. Suggestions to improve IDSR included pre-service and community training, mentorship, regular supervision and improving funding at the district level.

**Conclusion:**

Health workers perceived that scaling up revitalized IDSR training in Uganda improved public health surveillance. However, they acknowledge encountering challenges that hinder their performance after the training. Ministry of Health should have a mentorship plan, integrate IDSR training in pre-service curricula and advocate for funding IDSR activities to address some of the gaps highlighted in this study.

## Background

The majority of countries on the African continent are still faced with a high burden of infectious diseases [1] Expansion of global transport networks and subsequent increase in speed and ease of travel has increased the risk of rapid spread of pathogens and vectors around world [2] All countries need to implement robust public health disease surveillance systems to combat this public health threat. The Integrated Disease Surveillance and Response (IDSR) strategy is a comprehensive approach adopted by the African Regional Office of the World Health Organization (WHO/AFRO) in 1998 to improve disease surveillance in the region [3, 4]. IDSR provides a framework for strengthening surveillance, response and laboratory core required by the revised International Health Regulation [IHR (2005)]. In turn the IHR regulations can serve as a driving force to sustain national commitment to surveillance. The ability to report events of potential public health concern is grounded on strength of the surveillance system capacities [5]. The IDSR strategy was adopted in Uganda in 2000 and implementation commenced the following year [6, 7]. While surveillance was enhanced for all diseases, IDSR specifically focused on outbreak-prone diseases, diseases targeted for elimination or eradication and other diseases or conditions of public health deemed to cause high morbidity and mortality [8]. Since the adoption of the IDSR strategy in Uganda in 2000, several interventions were implemented to improve performance. These interventions targeted strengthening of IDSR core activities (i.e., case-patient detection, confirmation, reporting, data analysis and interpretation, response, feedback and dissemination) and support functions (i.e., training, communication, coordination, supervision, and resources). Initially, these efforts resulted in marked improvements in most IDSR core indicators, including completeness and timeliness of reporting, the proportion of outbreaks investigated within 48 h and reduction in case fatality rates for epidemic diseases such as cholera [9]. Previous studies on perception of health workers about new programs mainly focused on community case management of childhood diseases among community health workers. The studies show positive perception and constraints with added responsibility to treat and respect in the community [10–12]. Several new developments in disease surveillance took place years after the assessment. The inception, in 2011, of the short message system (SMS) reporting platform, codenamed *m-Track*, replaced the legacy system for transmitting weekly surveillance data through traditional channels such as physical delivery and email. In 2012 Uganda MOH in collaboration with WHO revised the IDSR technical guidelines according to IHR (2005), new concepts such as One Health; the increasing burden of non-communicable diseases (NCDs), maternal child health event and avian influenza. In Uganda, frontline health worker IDSR training was scaled up in Uganda since 2014 to enhance the capacity to detect, report and respond in time to disease epidemics and other public health events [13]. In 2009, the Uganda Ministry of Health (MoH) conducted an assessment on the implementation of the International Health Regulations (IHR 2005) however there is limited literature on perception of training IDSR support function [9, 14, 15]. While improvement of IDSR core indicators was earlier documented, understanding health worker behaviour is key to improvement in performance. In this study, we present findings of a cross-sectional qualitative study that explored perceptions of health care workers regarding the IDSR. Specific objectives included assess benefits, constraints and suggestion for improvement of the training.

## Methods

### Study context

Ministry of Health and National level institutions referral including national referral hospitals are mandated to lead health service delivery including public health emergencies at national level. At this level policy are made, resources allocated, with regards to surveillance they report events of public health concern to World Health Organization and the national task force which is a multisectoral organ coordinates response to public health events.

In Uganda health service delivery has been decentralized to district and Health sub district levels (HSD). The district has a responsibility of managing human resource for health, monitoring and supervision. HSD level is mandated with planning and managing health service delivery at lower health facilities within its catchment area. HSD has a referral facility which is either hospital or health centre IV. With regards to surveillance HDS receives monthly surveillance reports forms from lower facilities and forward aggregated report to district level.

Health facility level includes all institutions (public, private, non-government organizations) offering both basic in-patient and out-patient services, whereas HC IIs are at the parish level and provide only out-patient services. The HC I has no physical structure but comprises a village health team(VHT) which links the community to the health system. Uganda is divided into 14 health regions of which 13 were trained by the time of the evaluation.

### Study design

This evaluation was a cross-sectional study which utilized the qualitative approach. We conducted Focus Group Discussions (FGDs) at health facility level. We considered FGDs with health workers to be more appropriate for generating information about IDSR training, and suggestions for improvement of training IDSR in Uganda.

### Data collection tools

Semi structure focus group discussion guide were developed and pre-tested in one of the districts. Discussion guides had open ended questions regarding of the benefits of IDSR training, aspects of the training that are not good, aspects of IDSR training that participants were not able to practice and recommendations to improve the training. The pre-test was followed by revision and re-testing of the tools before the commencement of evaluation. The district where pretesting was done was excluded from the evaluation.

### Sampling methods

**Fig. 1:**
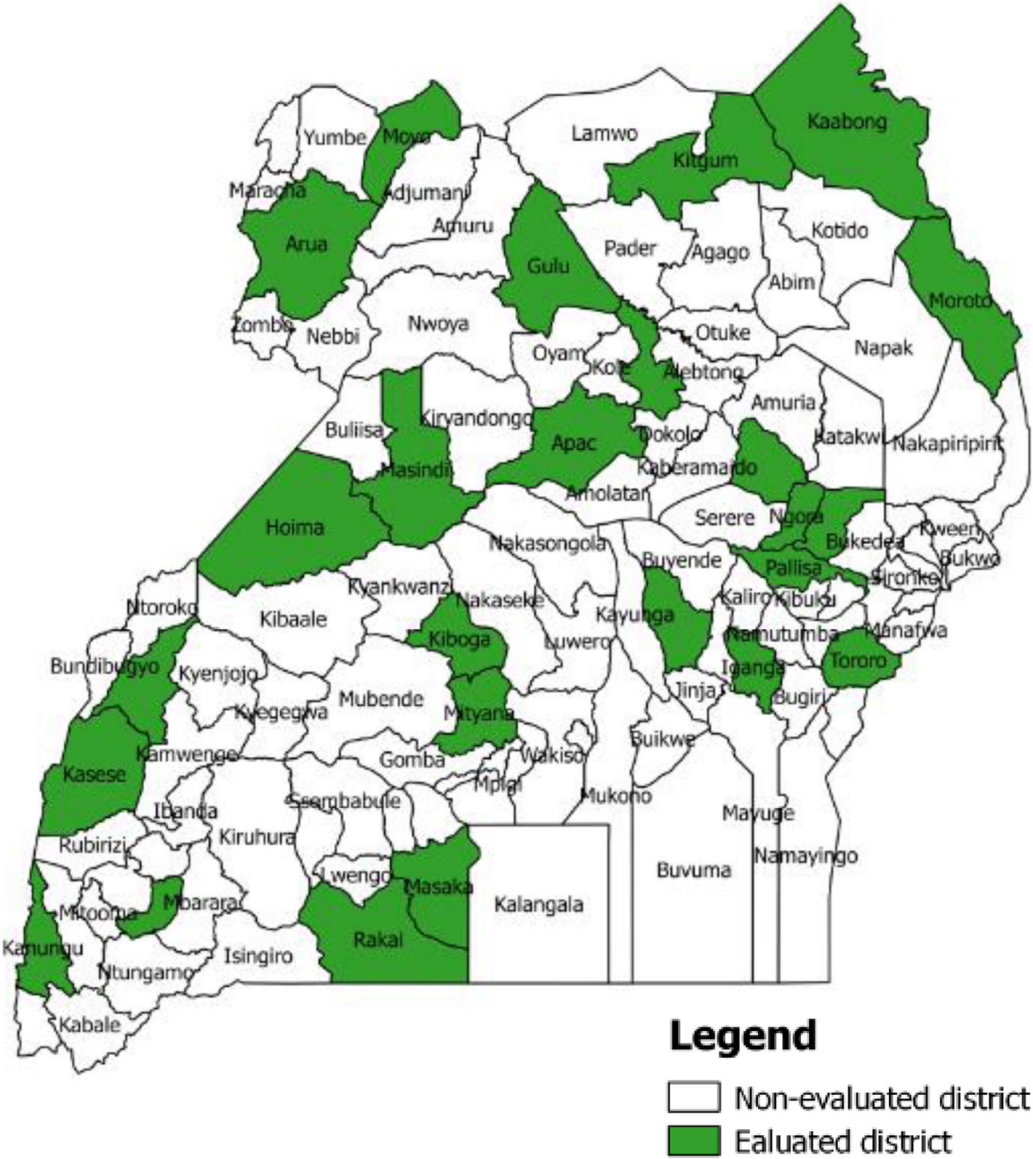
The map of Uganda showing districts selected for IDSR Revitalization Evaluation, 2016

We used multistage sampling using sampling frames with all districts per health region, purposively selected 2 districts with either hospital or a health centre IV (Fig. 1) and randomly selected health facilities where interviews were conducted. Districts sampled included five urban and 17 rural. All districts in each region were trained at the same time with average time to interviews of nine months. Five to ten health workers, at least three of whom were trained in IDSR were selected from each hospital or health centre to participate in the focus group discussion. Interviewers ensured representation from different departments. In-Charges of hospitals were excluded from FGDs to allow for free discussions amongst their staff.

### Data collection

Two independent trained interviewers conducted the interviews, on average lasting for one hour. The district staff assisted the research team mobilise participants from selected health facilities at an appointed time. The discussions took place in available space at the health facility such as meeting or training rooms for. We collected data between 19th June 2016 and 1st July 2016. On each of the FGD questions, interviewers probed the respondents to explain or elaborate their responses.

### Data analysis

Discussions were recorded, transcribed, translated and coded using grounded theory. Epi-Info version 7 United States, Centers for Disease Control Prevention (US CDC) was used to generate the frequency of coded issues for each of the FGD question.

### Ethical consideration

This research was done as part of an evaluation of IDSR performance by the Ministry of Health Uganda led by the Epidemiology and surveillance division. The study was requested by the Ministry of health as part of an evaluation of the surveillance system (IDSR). We obtained verbal consent from the participants. The consent procedure was approved by the National Task Force on Epidemics and Emergencies of Ministry of Health Uganda with technical support from Uganda World Health Organization Office.

## Results

### Characteristics of FGD participants and key informants

A total of 22/26 FGDs involving 216 health workers were conducted (Table 1). The majority of health workers who participated in FGDs were females (58%); their overall mean age was 38.8 years, and the majority (47%; *n* = 101) were nurses.

**Table 1:** Characteristics of Focus Group Discussions and participants (*N* = 22)

Characteristic	Region				Total
	Central	Eastern	Northern	Western	
Gender					
Male	17	21	25	27	91
Female	20	32	36	38	125
Mean age of FGD participants	38.3	40.3	39.5	38.1	38.8
Designation of FGD participants					
Nurse	10	32	28	31	101
Clinician	6	7	8	11	32
Laboratory	9	5	12	9	35
Medical Records Officer	9	6	7	9	31
Other	3	3	6	5	17

### Benefits of IDSR training at health facility level

The most commonly cited benefits of the training were its contribution to improved completeness and timeliness of reporting (Table 2, issue 1.1–1.2). According to participants, the training increased their awareness and changed their attitudes about the importance of disease surveillance. Also, trainees were taught how to do the weekly HMIS reporting via mTrac (a mobile telephone text message-based reporting system) which eased reporting from health facilities:

*Before we were trained in disease surveillance, many of us never took surveillance to be important. After the training, our attitude towards surveillance completely changed and we are now able to compile and submit all our weekly and monthly reports to the district without fail (FGD participant, Moyo Hospital).*

*We used to compile our weekly report and physically deliver it to the District Health Officer’s office which is more than 30 km away. This made it very difficult to beat reporting deadlines set by the ministry of health, and sometimes the reports were not delivered. However, ever since mTrac was rolled out in the district, reporting has become much easier since we are now able to submit weekly reports by use of mobile telephones (FGD participant, Kalisizo Hospital).*

**Table 2:** Assessment of IDSR training by Focus Group Discussion participants

Theme (aspect of IDSR training)	Key issue
1.0 Benefits of the training	1.1 Completeness of reporting has improved 1.2 Timeliness of reporting has improved 1.3 Improvement in case detection 1.4 There is better response to outbreaks 1.5 Data analysis has improved
2.0 Aspects of the training that are not good	2.1 Few health workers were trained 2.2 The training duration was inadequate 2.3 No follow-up or supervision was done after the training 2.4 No IDSR refresher training was done 2.5 No PPE/IPC materials were supplied
3.0 IDSR training aspects not practised	3.1 IDSR supervision and mentorship 3.2 Active case search 3.3 IDSR review meetings or CPDs
4.0 Recommendations to improve future IDSR training	4.1 Train more health workers 4.2 Post-training supervision and mentorship 4.3 Increase the duration of the training 4.4 IDSR training should be conducted regularly 4.5 Train community members in IDSR 4.6 Integrating IDSR into pre-service training

Improvement in case detection and better response to disease outbreaks were also commonly mentioned by participants (Table 2, issue 1.3 and 1.5):

*A few months ago, we had a yellow fever outbreak in our district which affected many people. Fortunately, this came at a time when the ministry of health had built our capacity in responding to disease outbreaks. We were able to investigate and respond to this outbreak (….) (FGD participant, Masaka Hospital).*

*(….) whenever there was an epidemic, Doctors would come from the ministry of health headquarters to handle the situation, but nowadays the district is much involved. We were trained on how to handle disease outbreaks and surveillance (….) (FGD participant, Gulu Hospital).*

In most of the FGDs, participants reported that data analysis has improved at departmental and hospital levels (Table 2, issue 1.4). This achievement was attributed to the training which empowered them with basic skills to conduct surveillance data analysis. However, some participants from lower health facilities acknowledged they were not utilising data analysis skills acquired.

### Negative aspects of IDSR training

When health workers were asked about what aspects of IDSR training were not good, there was a general agreement that the number of health workers trained was very small; this was mentioned in 54% of FGDs (Table 2, issue 2.1):

*(….) we are over forty health workers involved in diagnosis and reporting of health conditions to the district and mTrac yet only about 10 staff were trained in IDSR. Having a large pool of trained health workers enables health facilities to report regularly and consistently (FGD participant, Itojo Hospital).*

Participants also expressed concern that the duration of IDSR training was inadequate. They reported that the short duration impacted negatively on the delivery of knowledge and skills to trainees (Table 2, issue 2.2):

*The training was only conducted for three days, yet the training materials to be delivered to health workers were so many, and this forced the facilitators to rush through sessions without giving us time to appreciate some of the issues (FGD participant, Iganga Hospital).*

Other participants pointed out that after the training, there was no follow-up supervision or refresher training conducted (Table 2, issues 2.3–2.4).

### Aspects taught during IDSR training but are not practised

When health workers were asked about aspects of IDSR that they were not able to practice in their day to day work, the most commonly cited aspects included: 1) IDSR supervision and mentorship; 2) active case search and 3) IDSR meetings (Table 2, issue 3.1–3.3).

Across several FGDs, participants pointed out that although IDSR support supervision and mentorship were emphasised during IDSR training, hospitals are unable to fulfil this requirement (Table 2, issue 3.1). In their opinion, hospitals are mandated to conduct IDSR support supervision and mentorship of health workers at lower health facilities:

*Due to lack of funds, it is very difficult to practice some of the elements of surveillance emphasised in training such as follow-up of cases, supervision, (….) (FGD participant, Moyo Hospital).*

While active case search is one of the key requirements for prompt detection of certain conditions under IDSR approach, participants indicated that this is not consistently implemented at health facility level (Table 2, issue 3.2). Some of the barriers to active case search included lack of funds and logistics, inadequate staffing and poor staff attitude. Participants suggested that health facilities should be provided with funds to conduct community active case search in their respective catchment areas.

The issue of not conducting IDSR Continuous Professional Development (CPD) or IDSR review meetings at health facility level was also reported by some participants during FGDs (Table 2, issue 3.3). Most participants attributed this weakness to heads of key hospital departments that are involved in the implementation of surveillance activities at health facilities.

### Suggestions to improve future IDSR training

Respondents identified six major recommendations to improve and make future IDSR training courses more relevant. These included: training of more health workers, conducting regular post-training IDSR supervision or mentorship, increasing the duration of the training, conducting regular IDSR training, training of community members in IDSR and integration of IDSR into pre-service training curricula for health workers (Table 2, issues 4.1–4.6).

Health workers appreciated the Ministry of Health’s initiative of building their capacity to address public health problems in their districts through IDSR training. However, there was a general agreement that those who were trained were very few and therefore more health workers should be trained in disease surveillance (Table 2, issue 4.1) to bridge knowledge gaps that still exist among health workers:

*(….) very few people were trained, and many of those that were trained have left. In my opinion, every health worker should have knowledge and skills to implement IDSR at their health facilities, and such skills can be acquired through training or mentorship (FGD participant, Arua Regional Referral Hospital).*

Participants pointed out that IDSR training should be supported by regular supervision and mentorship of health workers at the centre of IDSR implementation (Table 2, issue 4.2). In discussions where this recommendation came up, there was a consensus among participants that supervision and mentorship is a key strategy that may address challenges related to lack of awareness and IDSR knowledge at health facilities:

*Regular supervision and mentorship are a necessity if we are to improve our performance and if the IDSR programme is to have an impact (FGD participant, Kaabong Hospital).*

*(….) mentorship will ensure that health workers that never had a chance to be trained are empowered with basic knowledge (....) (FGD participant, Pallisa Hospital).*

*We have several new staff who don’t know much about IDSR and some staff who were trained in surveillance have left (FGD participant, Kaabong Hospital).*

By design, health workers at health facility level were trained on IDSR for 3 days. However, this duration was deemed to be too short by many FGD participants. Participants recommended the IDSR training duration should be increased from the current three days to five or seven days (Table 2, issue 4.3).

*The training materials were too many to be delivered in the three days allocated for IDSR training. I suggest that the training duration should be increased from three days to seven days to enable participants to enable a better appreciation of all the materials in the training package (FGD participant, Kamuli Hospital).*

While the majority of respondents strongly agreed that training of more health workers, post-training supervision and mentorship would greatly improve IDSR performance, others believed that having regular IDSR training course was quite essential for making the IDSR programme a success (Table 2, issue 4.4).

*The recent IDSR training was conducted more than 5 years after we received the first training. Adults forget fast, and it is important that we have regular training to remind ourselves about surveillance and how to manage outbreaks (....) (FGD participant, Hoima Hospital).*

*(….) routine training in surveillance will ensure that new health workers are oriented in surveillance (FGD participant, Arua Hospital).*

There was also an agreement among participants that community members should be trained in IDSR. However, a few participants argued that existing Village Health Teams (VHTs) may not be reliable in conducting community surveillance since they are implementing several public health programs.

Although the recommendation to incorporate IDSR into pre-service curricula was not commonly cited (Table 2, issue 4.6), participants pointed out that this strategy will ensure that all health workers are empowered with basic skills and knowledge about disease surveillance and outbreak response:

*Disease surveillance is part and parcel of our routine work yet during my training as a nurse; we never had any training module on surveillance. I learnt about IDSR after starting work a few years ago. Disease surveillance should be included in training curricula to ensure that all health workers have basic knowledge (….) (FGD participant, Soroti Hospital).*

*Although it is important to have on-job IDSR training, IDSR should also be emphasised in all institutions that are involved in the training of different cadres of health workers (FGD participant, Kitagata Hospital).*

## Discussion

The findings of this study suggest that the IDSR training within the revitalised IDSR programme led to positive changes in public health surveillance in Uganda. Our findings indicate that participants perceived an improvement in timeliness and completeness of reporting. On the other hand, participants expressed the need for further trainings to understand all IDSR aspects, support supervision and continuous professional improvement meetings are needed to improve IDSR.

Our findings indicate that participants perceived an improvement in timeliness and completeness of reporting. This finding could be explained by the introduction of mTrac, national specimen collection and transportation system, the appointment of surveillance focal persons at district and health facility levels. This is finding is consistent with reports of improvement in indicators, such as completeness and timeliness of reporting, case detection and response to disease outbreaks, following implementation of IDSR [9, 16–18]. Evaluations carried out since the introduction of IDSR implementation in Uganda have revealed fluctuations in the performance of IDSR core performance indicators. National level completeness of reporting was 85% in 2007 and 83% in 2009 compared to 77% observed in the 3 months preceding this study. Whereas the national level timeliness of reporting was 53% in 2007, this improved to 68% in 2009 and remained at 68% in 2016 when this study was conducted [9, 14] The fluctuation in performance could be explained by the creation of new districts which lacked the technical capacity to implement IDSR [9]. By the time Uganda started implementation of IDSR strategy in 2001, it had 56 districts, but at the time this study was conducted the country had 112 districts. This finding is consistent with earlier studies, and it contributes to underperformance and decline in performance [9, 19]. The respondents mentioned the introduction of mTrac and regular supply and availability of reporting tools at health facility level which has improved case detection. Mtrac is a short phone message sent to Health Management Information System (HMIS) to alert higher level of any suspected case detected by health workers. Health workers mentioned that the training empowered them to respond to outbreaks at district level before the national teams reach. In this study, we found that 88% of health facilities had weekly and monthly epidemiological surveillance reports. Another study conducted in neighbouring Kenya reported the availability of data reporting forms as a contributing to adequate weekly surveillance reporting [20, 21].

In this study, the participants had a perception of improvement in data analysis after IDSR training. Training provides a standard data analysis format thus improving data use for decision making. This is consistent with earlier studies in Nigeria and four other African countries where training provided guidance on data analysis and improved performance [15, 21]. Participants pointed out that although IDSR support supervision and mentorship were emphasised during IDSR training, hospitals are unable to fulfil this requirement. This finding could be explained by lack of funding for surveillance activities at all district hospitals. Adokia et al. also had similar findings from Ghana [22].

Whereas the revitalised IDSR programme in Uganda was viewed as successful, the new focus should now be on how to address the current challenges to improve IDSR performance and sustain these achievements. In this study participants suggested IDSR training could be improved by increasing the number of participants and days for IDSR training, suppport supervision and mentorship, refresher trainings for in-service trainees, involve community health workers and include IDSR in pre-service training.

Regular training was cited as another way to improve IDSR training for in-service trainees. Refreshing of IDSR knowledge of new health workers that have joined the health sector is important because it prepares them to solve any surveillance problems encountered. Refresher training serves as a reminder of the IDSR guidelines. There was a consensus that increasing the duration of the training will enable health workers to appreciate the concepts of surveillance much better:

In this study supervision and mentorship were sighted to improve health workers performance in IDSR. Irregular IDSR supervision was highlighted as a key challenge affecting public health surveillance in our study and other studies in Uganda and Ghana [23, 24]. It is essential that regular support supervision complements training courses, and IDSR mentorship activities at health facility level. Previous experience in Uganda and elsewhere shows that training is associated with improvement in IDSR indicators.

Village Health Teams (VHTs) as participants in IDSR training will improve community capacity for early detection of cases. VHTs have a great experience and a big role in the implementation of public health programs across the country. Training VHTs also improves community participation and increases their confidence in the health system.

While participants did not commonly express the issue of incorporation of IDSR training into pre-service training curricula for health training institutions, this strategy is very important in effectively addressing gaps in the sustainability of trained human resources. This is because the current strategy of in-service training is already hampered by insufficient numbers of trained medical personnel and high turnover of staff. The second challenge of the in-service training programme is related to high costs involved in the training of health workers and sustainability of the programme. IDSR training does not have budget line in Ministry of Health because it is funded by implementing partners. A study done in Ghana revealed that collaboration with public health schools helped institutionalize IDSR training in the basic medical training, ensuring sustainability [25]. This study was implemented as part of the large evaluation of IDSR functions and could have limited time to have indepth interviews to better understand perceptions. However, this study provided insight into health workers perceptions which could be used for further quantitative studies.

## Recommendations

The MOH and its partners should consider developing an in-service training plan that is based on the needs of health workers, who are key implementers of the IDSR programme. To effectively bridge knowledge gaps at implementation levels, IDSR training should target a larger number of in service health workers in the public and private sectors. The Ministry of Health should have a mentorship plan to enhance IDSR skills of health workers after the trainings. Integration of the IDSR training in pre-service curricula for health workers is a sustainable strategy that should be considered to address some of the gaps highlighted in this study.

## Conclusion

Front line health workers perceived IDSR training to have led to an improved IDSR performance at health facility level. Training frontline health workers is having a robust public health surveillance system with capability for prevention, early detection and response. Mentorship and support supervision were other options cited to improve IDSR performance among in-service health workers. However, successful implementation of the IDSR programme in Uganda requires that the Ministry of Health and its partners address knowledge gaps at health facility level.

## Abbreviations

CERF: Central Emergency Response Fund
CPD: Continuous Professional Development
FGD: Focus Group Discussion
HMIS: Health Management Information System
IDSR: Integrated Disease Surveillance and Response
IHR: International Health Regulation
MOH: Ministry of Health
NCDs: Non-communicable diseases
RMANCH: Reproductive Maternal Newborn Adolescent and Child Health
UNICEF: United Nations Children’s Fund is a United Nations
US CDC: United States Centers for Disease Control Prevention
USAID: United States Agency for International Development
VHTs: Village Health Teams
WHO AFRO: World Health Organisation Africa Regional Office
